# Impact of topical applications of sunflower seed oil on neonatal mortality and morbidity in southern Nepal: a community-based, cluster-randomised trial

**DOI:** 10.1136/bmjgh-2023-013691

**Published:** 2024-02-29

**Authors:** Joanne Katz, Subarna K Khatry, Laxman Shrestha, Aimee Summers, Marty O Visscher, Jeevan B Sherchand, James M Tielsch, Seema Subedi, Steven C LeClerq, Luke C Mullany

**Affiliations:** 1 Department of International Health, Johns Hopkins Bloomberg School of Public Health, Baltimore, Maryland, USA; 2 Nepal Nutrition Intervention Project, Kathmandu, Nepal; 3 Institute of Medicine, Tribhuvan University, Kirtipur, Nepal; 4 Cincinnati Children’s Hospital Medical Center, Cincinnati, Ohio, USA; 5 Tribhuvan University, Kirtipur, Nepal; 6 Global Health, George Washington University School of Public Health and Health Services, Washington, District of Columbia, USA

**Keywords:** Child health, Paediatrics, Cluster randomized trial

## Abstract

**Introduction:**

Hospital-based studies have demonstrated topical applications of sunflower seed oil (SSO) to skin of preterm infants can reduce nosocomial infections and improve survival. In South Asia, replacing traditional mustard with SSO might have similar benefits.

**Methods:**

340 communities in Sarlahi, Nepal were randomised to use mustard oil (MO) or SSO for community practice of daily newborn massage. Women were provided oil in late pregnancy and the first month post partum, and visited daily through the first week of life to encourage massage practice. A separate data collection team visited on days 1, 3, 7, 10, 14, 21 and 28 to record vital status and assess serious bacterial infection.

**Results:**

Between November 2010 and January 2017, we enrolled 39 479 pregnancies. 32 114 live births were analysed. Neonatal mortality rates (NMRs) were 31.8/1000 (520 deaths, 16 327 births) and 30.5/1000 (478 deaths, 15 676 births) in control and intervention, respectively (relative risk (RR)=0.95, 95% CI: 0.84, 1.08). Among preterm births, NMR was 90.4/1000 (229 deaths, 2533 births) and 79.2/1000 (188 deaths, 2373 births) in control and intervention, respectively (RR=0.88; 95% CI: 0.74, 1.05). Among preterm births <34 weeks, the RR was 0.83 (95% CI: 0.67, 1.02). No statistically significant differences were observed in incidence of serious bacterial infection.

**Conclusions:**

We did not find any neonatal mortality or morbidity benefit of using SSO instead of MO as emollient therapy in the early neonatal period. Further studies examining whether very preterm babies may benefit are warranted.

**Trial registration number:**

ClinicalTrials.gov Registry (NCT01177111).

**WHAT IS ALREADY KNOWN ON THIS TOPIC**

- Regular and repeated oil massage of newborns with mustard seed oil in South Asia is an ubiquitous practice, but mustard seed oil has been shown to have a negative effect on the skin in animal studies.
- Sunflower seed oil, which is high in linoleic acid, may reduce morbidity and mortality in neonates compared with mustard seed oil.
- One community-based randomised trial in Uttar Pradesh, India did not find an overall mortality benefit with sunflower seed oil massage compared with usual practice in study communities but did find a benefit among very low birthweight infants.

**WHAT THIS STUDY ADDS**

- Communities in southern Nepal were randomised to receive either mustard or sunflower seed oil massage with neonatal morbidity and mortality as the outcome.
- Unlike the trial in India, the one in Nepal did not attempt to change the traditional massage technique or oil quality, but only aimed to compare the two types of oils.
- As with the trial in India, this study found no overall morbidity or mortality benefit but found that preterm infants, especially very preterm (<34 weeks’ gestation), had higher survival rates in the sunflower seed oil massage group, although the differences were not statistically significant.

**HOW THIS STUDY MIGHT AFFECT RESEARCH, PRACTICE OR POLICY**

- The results of these two community-based trials do not suggest an overall impact of switching oil massage practices from mustard to sunflower seed oil, regardless of whether the massage technique is changed to make it less vigorous.
- However, both trials suggest that very small or very preterm infants may benefit from switching oils, and confirmatory studies should be done in these vulnerable groups in community settings.

## Introduction

Neonatal deaths account for almost half of all child deaths; in 2019, there were approximately 2.44 million deaths, with the vast majority occurring in low and middle-income countries.^1^ A main cause of death is complications arising from preterm birth (36% of neonatal deaths), and options for appropriately managing babies born early are limited.^1 2^ In the past 20 years, there has been increased recognition of the important role of skin in neonatal survival and protection from infection. One skin-related intervention is the use of emollient therapy with natural vegetable oils, but most studies of this intervention have focused on prevention of infection rather than survival.^3–7^

Emollient therapy for reducing infection and improving survival of preterm babies has largely focused on improving the care of babies in intensive care units or hospitalised settings.^5–9^ Efficacy appeared mixed, and meta-analysis and systematic reviews in some studies showed that nosocomial infection risk was not reduced, and others showed reductions in infection and neonatal mortality.^3 4 10 11^

Further, studies in animal models of the varying impact of commonly used natural vegetable oils in mice suggested that one of the most used oils, mustard seed oil, was perhaps the least appropriate for skin recovery after massage, while exposure to sunflower seed oil appeared to mitigate these effects and possibly accelerate skin barrier function.^12^ These observations, in particular, the demonstration of accelerated skin recovery after application of high-linoleic acid sunflower seed oil, and its ability to enhance skin barrier function, motivated the design of hospital-based randomised trials of repeated massage with sunflower seed oil in Egypt and Bangladesh.^5 6^ In Egypt, infants (n=51) receiving sunflower seed oil applications had improved skin condition and 44% (19–74%) had lower risk of nosocomial infections compared with controls (n=52). In a much larger trial in Bangladesh, sunflower seed oil again improved skin condition, reduced nosocomial infections (N=497) (RR=0.59 (0.37, 0.96)) and mortality (relative risk (RR)=0.74 (0.55, 0.99)) compared with controls.^8^ However, these trials were all done in hospital settings with preterm infants.

These findings raised the possibility that in communities where mothers and other caretakers provided daily, repeated full body massage of their newborn infants with mustard seed oil, a switch to a high-linoleic oil such as sunflower seed oil might reduce infection risk and improve survival, especially among preterm babies.^13 14^ Traditional practices often include vigorous massage of the newborn that could damage skin or remove protective vernix. The impact of a cluster-randomised trial in a community-based setting comparing neonatal massage with mustard with sunflower seed oil on neonatal mortality from 2014 to 2016 in Uttar Pradesh, India was recently published.^15^ This trial compared standard of care oil massage with gentle massage three times per day with sunflower seed oil. While mustard oil was the single most commonly used oil in the standard of care group, only 36% of newborn infants in the comparison group used mustard oil exclusively.^15^ The trial found no difference in neonatal mortality between the two groups (adjusted OR=0.96 (0.84, 1.11)) but a reduction in mortality among the 2.2% of infants weighing <1500 g (adjusted OR=0.48 (0.26, 0.88)).

We had previously shown that among mother/newborn dyads in communities of rural Sarlahi District, Nepal, oil massage was widespread (ie, among >99% of babies) using a range of locally available mustard oils.^13^ We further learnt through qualitative interviews and querying acceptability and preferences related to oil massage that recently delivered women would consider trying an alternative oil. We thereafter designed and now report results of a large community-based cluster-randomised trial to examine the impact of switching from mustard to sunflower seed oil for newborn massage without attempting to change the massage frequency or technique, on all-cause mortality, mortality among preterm babies, and signs of infection in the days and weeks after birth.

## Methods

### Setting and study population

The study population for this project consisted of liveborn infants born in households in participating Village Development Committees (VDCs) in Sarlahi District of low-lying southern Nepal. Prior to decentralisation, Nepal was divided into zones, districts within zones and VDCs within districts; we retain this language to describe the administrative geographies, as this study was designed and implemented prior to decentralisation. Sarlahi District lies along the border with Bihar State in northern India and is typical of most of northern India and large parts of western Bangladesh and southern and central Pakistan. Over the past 30 years, the Nepal Nutrition Intervention Project-Sarlahi has conducted research predominately in the northern portion of the district. All pre-decentralisation VDCs were divided into nine wards (a government unit). We further divided wards into one or more sectors based on population size. These sectors were the unit (cluster) of randomisation in this study. Each of these sectors was monitored by a local resident woman, whom we called a ‘ward volunteer’ or ‘WV’. We initially selected 13 VDCs to participate in the study; this number was expanded in 2014 from 13 to 34, to increase the rate of enrolment (see the Sample size and Data safety and monitoring sections).

The population in the study area was comprised primarily of subsistence farmers, mostly Hindus, with smaller subgroups of Buddhists, Muslims, Christians and animists. Prior to our study, about 30% of households were landless, 76% had no electricity and overall socioeconomic status in the population varied minimally. Sarlahi is considered a poor area, even in Nepal; only 37% of male heads of household and 6% of female heads of households were literate at study inception, and 75% of the population lived below the poverty line established by the government of Nepal (https://www.macrotrends.net/countries/NPL/nepal/poverty-rate). At the time of initiating the study, the total population in the proposed VDCs was approximately 300 000, the annual birth cohort was approximately 7000 infants and the neonatal mortality rate (NMR) was approximately 30/1000 live births.

### Study design and intervention

Our study was a cluster-randomised, community-based trial in Sarlahi District of southern Nepal. Newborn babies were randomised within geographical clusters (sectors) to receive either (1) provision of and promotion of use of sunflower seed oil, or (2) provision of and promotion of use of mustard seed oil for neonatal massage, with massage to be carried out according to the caretaker/household normal practice.

### Randomisation and blinding

Each of the 9 wards in the 34 participating VDCs were divided into one or more sectors. On average, there were 10 sectors per VDC. A locally residing female worker assigned to each ward (WV) was responsible for identification, tracking, and following mothers and babies within their specific area. As the oil allocation was done at this level, each individual WV only promoted one type of oil. We chose to randomise sectors rather than individual pregnant women/baby dyads to minimise the likelihood of intervention crossovers. The actual allocation was done using two separate processes. The first was for the initial 13 VDCs, which included 136 WV areas (clusters). A computerised quasi-random number generation procedure was used to randomise clusters to either mustard oil or sunflower seed oil. The randomisation was restricted, using tertiles of prior estimates of neonatal mortality, to ensure balance on prestudy mortality risk. In this original randomisation, 68 clusters were assigned to each of the groups. For the expansion of VDCs (n=21 VDCs, 205 clusters), we did not have prior mortality data and instead restricted on the cluster-specific number of married women of reproductive age. Sector assignment lists were maintained at study headquarters in Kathmandu and Sarlahi. WVs were informed of their sectors’ allocation to either sunflower seed oil or mustard seed oil, and they were consistently provided that oil to promote and distribute throughout the course of the study. Participants, field workers and investigators were not blinded to the allocation as the oils had different appearances and smells.

### Study procedures and data collection

All study procedures, data collection contact points and information collected were identical in both study arms, except for the type of oil that was distributed and promoted (ie, sunflower seed oil, mustard seed oil).

Pregnancy identification: we first updated our research site’s existing sector-specific maps and household structure database by visiting each household and collecting basic information on all women of reproductive age. This information was used by locally residing female project workers to monitor a universal sample of married women of reproductive age for incident pregnancies. All women were visited at home every 5 weeks and asked about menstruation in the prior 5 weeks and offered pregnancy tests if they had not menstruated. Date of last menstrual period (LMP) was recorded at this time, hence early in pregnancy rather than at time of delivery. Gestational age was calculated as time between date of LMP and date of birth. All newly married women in the community were added to this list to maintain a universal list and to maximise early identification of pregnancies, which were reported to a supervisory staff member for consent and enrolment into the main trial.

### Enrolment of pregnant women

Workers approached identified pregnant women at home, explained the study in full and obtained verbal consent for participation. Women provided standard demographic information and reproductive history, and household socioeconomic status. A set of basic interventions was provided to all women, to meet recommendations for essential newborn care as part of the standard Nepal Neonatal Health Strategy. These basic interventions included a clean delivery kit containing soap for birth attendant hand-washing, a sterile blade and cord tie, and a plastic sheet for a clean delivery surface, 90 days of iron folate supplements and deworming medication (if these had not been received through the local health system), 4.0% chlorhexidine gel for cord cleansing (Lomus Pharmaceuticals), and promotional messages on maternal nutrition during pregnancy, danger signs and associated care-seeking, early and exclusive breast feeding, clean and hygienic delivery including cord care, hand-washing, and thermal care of the newborn. At monthly intervals between enrolment and pregnancy outcome, a field worker visited the household of the woman to record pregnancy status (ie, still pregnant or outcome had occurred) and to ask some basic questions about signs of morbidity during the previous 30-day period. At these visits, women also had their weight and blood pressure/pulse measured, and body temperature recorded. Women reporting signs of morbidity and indicating that these signs were currently present were referred to the local health post or primary health centre. Women with fever or elevated blood pressure as measured by our staff were similarly referred.

### Intervention

#### Oil acquisition, handling and content analysis

We acquired high-quality sunflower seed and mustard seed oils from Shiv Shakti Udyog, Jitpur, Nepal; the manufacturer is located approximately 60 km to the west of our study site. The company provided a date of manufacturing imprint on each individual sealed package and a recommended ‘use-by’ date (6–12 months). The company’s vegetable oil products are approved for a range of common uses including eating, cooking and topical therapy, and are representative of those found in open markets in rural areas of Nepal. This company, and all similar companies producing edible vegetable oils for commercial purposes in Nepal, submit samples for purity/quality to the government of Nepal Food Inspection Laboratory in Hetauda, Makwanpur District, Nepal (approximately 90 km to the west of our study site). Both oil types were purchased directly from the factory in 1 L bottles or 500 mL sealed plastic pouches (as sold on the market) and were stored at our site headquarters at room temperature; we purchased batches approximately every 4–6 weeks to minimise time between purchase and distribution. On a weekly basis, we used these stock batches to prepare small 100 mL and 500 mL bottles with a study-specific customised logo, indicating its use for baby massage. At semiregular intervals (every 6–12 months) throughout the study, we sent randomly selected samples of sunflower and mustard oil to Geo-Chem Laboratories (Mumbai, India) for basic fatty acid profile, including content (%) of linoleic, linolenic, palmitic, stearic, oleic and erucic acids.

#### Provision of oil and messaging to pregnant women

The oil was provided to pregnant women and resupplied throughout the neonatal period, along with promotional message to use it for the traditional practice of baby massage. Specifically, using the estimate of LMP collected at enrolment, we scheduled a home visit for approximately 28–30 weeks’ gestation; at this visit, a project field worker provided either sunflower seed oil or mustard seed oil, depending on the sector allocation, and imparted promotional messages regarding the use of this provided oil for the strict purpose of newborn massage throughout the newborn period. Each participating woman received an initial allocation of one 100 mL bottle and a small plastic finger bowl. The worker again explained the purpose of the study and discussed the potential benefits of newborn massage (ie, non-specific to choice of oil), including thermoregulation, improved mother and infant bonding through tactile stimulation, and absorption of essential lipids. The worker instructed the mother to reserve the use of the provided oil for massage of the newborn and encouraged initiation, timing and frequency of massage according to common practice. The worker shared messages with other family members, including grandmother or father of the newborn, during this visit.

After birth, the mother and/or other caretakers initiated the full body massage using the provided oil. The practice continued daily throughout the newborn period and followed the common practice in this community of two to three massages per day. While the topical application of the oil was provided by family members, the locally residing female worker visited daily during the first week of life to promote continued use of the provided oil; at each of these seven visits, she recorded the date, the number of times the newborn received a massage since her previous visit (as reported by caretakers) and which oil was used for massage (study provided or other).

#### Post-delivery data collection

Immediately after conducting the first of these daily home visits, the WV also notified a member of the Birth Assessment Team. These workers then visited the home as soon as possible to record information directly related to both mother and newborn including self-reported late pregnancy morbidity, characteristics of and circumstances surrounding labour and delivery, and direct measurements of maternal temperature, as well as infant characteristics such as date and time of birth, sex, length, weight and immediate newborn practices (thermal care, cord practices, early bathing and massage, breastfeeding initiation, etc). Weight was measured using a digital neonatal weight scale with precision to 10 g (Tanita BD-585). At this, and six subsequent home visits, scheduled for days 3, 7, 10, 14, 21 and 28 after delivery, the worker collected three further sets of information: first, the infant was examined for respiratory rate (breaths/min measured using a digital timer), axillary temperature (measured using a digital thermometer), signs of cord infection, chest indrawing, jaundice and skin infection. Second, maternal or other caretaker reports of signs of morbidity occurring since the birth (or since the prior visit, for subsequent contacts) including difficulty breathing, diarrhoea, convulsions, perceived fever, etc. Third, the worker recorded newborn care practices since the prior visit including breast feeding and other feeding practices, and questions regarding newborn massage, including the number of massages, timing and use of the project-provided oil. These workers also removed (on day 1) the 100 mL oil bottle provided at the late-in-pregnancy visit, replacing it with a fresh 500 mL bottle; these larger bottles were replaced on day 10 and day 21, ensuring that the mother always had an adequate supply of the project-provided oil aligned with her geographical cluster. At each visit, the worker recorded the approximate volume remaining in the bottles.

### Definition and measurement of outcomes

Preterm birth was defined as <37 completed weeks’ gestation. Moderately to very preterm birth was defined as <34 completed weeks’ gestation. Low birth weight (LBW) was defined as weight <2500 g and very low birth weight as <1500 g. Since weights were taken at varying times after birth, due to differences in the time it took the study team to reach the home after birth, we included only weights taken within 72 hours of birth in our analysis. Small for gestational age (SGA) was defined as having a birth weight below the 10th percentile of the Intergrowth reference population for each gestational age in days and sex of the infant.^16^ Severely ill newborns were defined using stringent criteria based on the presence of two or more of the following danger signs at any time in the neonatal period: caregiver’s report of the baby having (1) difficulty feeding or sucking; (2) difficulty breathing; (3) convulsions or stiffness of the back; or the trained field staff’s observation during newborn assessment of the baby having (4) severe chest indrawing; (5) rapid breathing (respiratory rate >70 breaths/min); (6) not moving upon stimulation; (7) umbilical cord infection (pus discharge from the cord stump or redness of the cord stump or around the base of the cord); (8) fever (axillary temperature to be ≥38°C or 100.4°F); or (9) hypothermia (axillary temperature to be <35°C or 95°F).

### Sample size

The sample size for this study was originally ~20 170 babies given calculation parameters estimated from data available at the time the study was originally proposed. That sample size, using a baseline NMR of 32/1000 live births, would have provided 80% power to detect a 20% reduction in neonatal mortality. However, in 2010, only partial funding was secured, decreasing the total number of babies that would be possible to enrol to approximately 10 500. This lower sample size provided 80% power only for reductions in mortality that substantially exceeded the initially proposed effect size (20%) thought to be of public health impact. Therefore, we continued to search for funding to expand the study back to a sample size that would provide power to detect lower effect size(s). In late 2013, we received funding from the Bill and Melinda Gates Foundation to expand our study site area from 13 VDCs to 34 VDCs, increasing the annual birth cohort to the numbers reported above, and allowing us to aim for an enrolment of 14 630 babies per group for a total size of 29 260. This new sample size requirement was based on a desired effect size of 20% for the primary outcome (all cause neonatal mortality) and used an updated estimate of control group mortality (28/1000). Although subgroup analysis among preterm infants was part of the original and expanded protocol, we did not power the study explicitly to answer this question. Our aim was to see whether this intervention could impact overall mortality in term and preterm neonates in the community, and if so, a programme would not need to be targeted to a subgroup.

### Analysis

The primary aims of the study were to determine if repeated topical applications of sunflower seed oil during traditional massage of newborns could reduce all-cause neonatal mortality risk by at least 20% and all-cause mortality risk among preterm babies by 25% compared with newborns receiving the current traditional massage using mustard oil. All analyses were conducted using Stata (Statacorp, College Station, Texas, USA) and R (R Core Team, 2019).

### Data safety and monitoring

The study was monitored by an independent Data Safety and Monitoring Board of five members, which met three times, once prior to initiating the study, once when the sample had reached ~50% anticipated enrolment size (total n=4791) and once when the sample had reached ~50% of the expanded enrolment size (total n=15 032). At both interim time points, the board examined enrolment progress, randomisation balance, intervention compliance, adverse events (morbidity, stillbirths, neonatal deaths, skin condition) and outcome data (neonatal mortality), and advised that enrolment continue as planned.

### Role of funders

The funders had no role in study design, data collection and analysis, decision to publish or preparation of the manuscript. A reflexivity statement outlining the role of our local research partners has been provided in the online supplemental material 1. This trial is registered at ClinicalTrials.gov (NCT01177111).

10.1136/bmjgh-2023-013691.supp1 Supplementary data

## Results

### Participants

**Figure 1:**
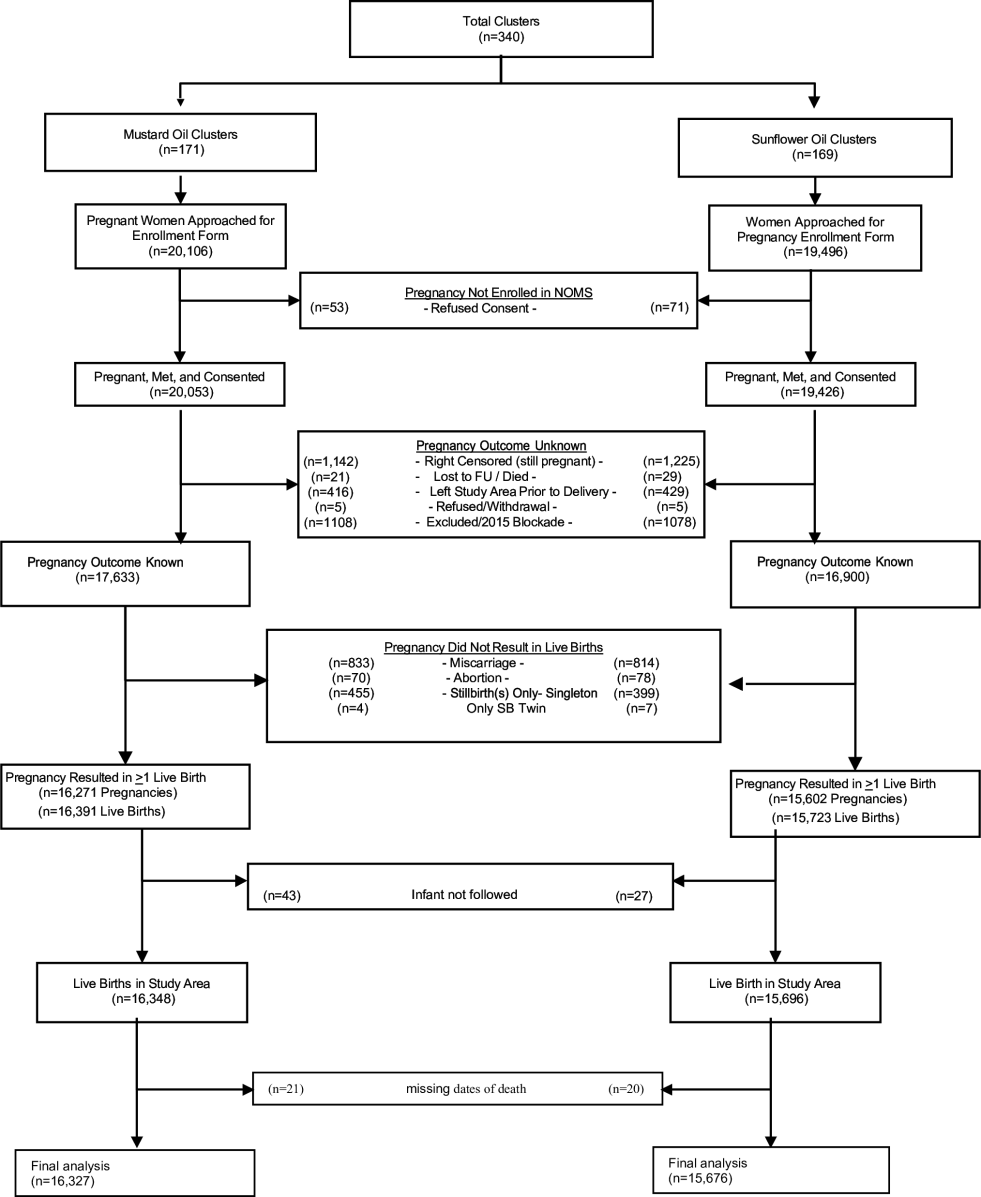
Consort Diagram for the Nepal Oil Massage Study (NOMS) trial. FU, follow-up.

A total of 340 geographical clusters were randomised to either mustard (n=171) or sunflower seed oil massage (n=169) (figure 1). From within these geographical areas, 39 479 women were enrolled in the trial. They contributed 34 533 pregnancies and 32 114 live births, 16 348 of whom lived in areas randomised to mustard seed oil massage and 15 696 of whom lived in areas randomised to sunflower seed oil massage.

### Baseline comparison

Infants in the two groups were comparable with each other based on household socioeconomic and demographic characteristics, maternal demographics, morbidity and health-seeking characteristics, and infant birth outcomes (sex, multiple births, gestational age, preterm, LBW and SGA) (table 1).

**Table 1:** Comparison of baseline characteristics of infants by intervention groups

Variables	Categories	Mustard oil	Sunflower oil
		N=16 327	N=15 676
		n (%)	n (%)
Household characteristics			
Ethnicity	Pahadi	667 (4.1)	639 (4.1)
	Madeshi	15 648 (95.8)	15 025 (95.8)
	Don’t know	1 (0.0)	2 (0.0)
	Missing	11 (0.1)	10 (0.1)
Any household ownership			
Mobile phone	No	1769 (10.8)	1772 (11.3)
	Yes	14 530 (89.0)	13 884 (88.6)
	Missing	28 (0.2)	20 (0.1)
Television	No	10 336 (63.3)	9807 (62.6)
	Yes	5978 (36.6)	5856 (37.4)
	Missing	13 (0.1)	13 (0.1)
Motorcycle	No	13 427 (82.2)	12 737 (81.3)
	Yes	2887 (17.7)	2927 (18.7)
	Missing	13 (0.1)	12 (0.1)
Latrine	No	11 778 (72.1)	11 151 (71.1)
	Yes	4535 (27.8)	4512 (28.8)
	Missing	14 (0.1)	13 (0.1)
Electricity	No	4341 (26.6)	4319 (27.6)
	Yes	11 969 (73.3)	11 340 (72.3)
	Missing	17 (0.1)	17 (0.1)
Agricultural land (khet)	No	6150 (37.7)	6104 (38.9)
	Yes	10 088 (61.8)	9493 (60.6)
	Missing	89 (0.5)	79 (0.5)
Kitchen garden (baari)	No	1048 (6.4)	1058 (6.7)
	Yes	15 159 (92.8)	14 511 (92.6)
	Missing	120 (0.7)	107 (0.7)
Maternal characteristics			
Age at LMP, mean (SD)		22.5 (4.7)	22.5 (4.8)
Age at LMP	<18	2454 (15.0)	2494 (15.9)
	18–35	13 536 (82.9)	12 814 (81.7)
	>35	337 (2.1)	366 (2.3)
	Missing	0 (0.0)	2 (0.0)
Height at enrolment in cm, mean (SD)		150.6 (5.5)	150.5 (5.5)
Height at enrolment	<145	2438 (14.9)	2265 (14.4)
	145–150	4811 (29.5)	4776 (30.5)
	≥150	9055 (55.5)	8608 (54.9)
	Missing	23 (0.1)	27 (0.2)
Enrolment weight in kg, mean (SD)		46.3 (6.9)	46.4 (7.0)
Education	None	11 095 (68.0)	10 462 (66.7)
	1–5 years	1401 (8.6)	1320 (8.4)
	>5 years	3814 (23.4)	3880 (24.8)
	Missing	17 (0.1)	14 (0.1)
Parity at enrolment	No prior pregnancy	4654 (28.5)	4544 (29.0)
	Prior pregnancy parity 0	402 (2.5)	389 (2.5)
	1–4	10 486 (64.2)	9958 (63.5)
	>4	694 (4.3)	704 (4.5)
	Missing	91 (0.6)	81 (0.5)
Prior deaths of any liveborn children	No prior pregnancy	4654 (28.5)	4544 (29.0)
	Prior pregnancies but no live births	560 (3.4)	521 (3.3)
	No deaths of prior live births	9001 (55.1)	8596 (54.8)
	Death of a prior live birth	1875 (11.5)	1766 (11.3)
	Missing	237 (1.5)	249 (1.6)
Number of ANC visits	None	2834 (17.4)	2725 (17.4)
	1–3	7170 (43.9)	6875 (43.9)
	≥4	4617 (28.3)	4349 (27.7)
	Missing	1706 (10.4)	1727 (11.0)
Delivered at a facility	No	8558 (52.4)	7931 (50.6)
	Yes	6089 (37.3)	6040 (38.5)
	Missing	1680 (10.3)	1705 (10.9)
Any delivery complications	No	11 541 (70.7)	11 023 (70.3)
	Yes	3078 (18.9)	2923 (18.6)
	Missing	1708 (10.5)	1730 (11.0)
Vaginal bleeding in 7 days before delivery	No	14 405 (88.2)	13 729 (87.5)
	Yes	240 (1.4)	234 (1.4)
	Missing	1682 (10.3)	1713 (10.9)
Fever with 7 days before delivery	No	14 287 (87.5)	13 677 (87.2)
	Yes	354 (2.1)	289 (1.8)
	Missing	1686 (10.3)	1710 (10.9)
Convulsions in 7 days before delivery	No	14 605 (89.4)	13 929 (88.8)
	Yes	39 (0.2)	40 (0.2)
	Missing	1683 (10.3)	1707 (10.8)
Infant characteristics at birth			
Sex	Female	7763 (47.5)	7409 (47.3)
	Male	8285 (50.7)	7995 (51.0)
	Twin/triplet	250 (1.5)	252 (1.6)
	Missing	29 (0.2)	20 (0.1)
GA at outcome (weeks), mean (SD)		39.4 (3.3)	39.4 (3.2)
Preterm	Term (≥37)	13 793 (84.5)	13 301 (84.8)
	Mild preterm (34–<37)	1805 (11.1)	1724 (11.0)
	Moderately preterm (<34)	728 (4.5)	649 (4.1)
	Missing	1 (0.0)	2 (0.0)
Birth weight taken within <72 hours, mean (SD)		2711.1 (427.6)	2704.1 (433.0)
LBW taken <72 hours of birth	No LBW	8694 (53.2)	8307 (53.0)
	LBW (<2500 g)	3713 (22.7)	3538 (22.6)
	Missing	3920 (24.0)	3831 (24.4)
SGA taken within <72 hours of birth, mean (SD)	No SGA	7683 (47.1)	7278 (46.4)
	SGA	6650 (40.7)	6409 (40.9)
	Missing	1994 (12.2)	1989 (12.7)
Delayed cord clamping	No	4352 (26.7)	4365 (27.8)
	Yes	9558 (58.5)	8959 (57.2)
	Missing	2417 (14.8)	2352 (15.0)
Immediate drying after delivery	No	8333 (51.0)	7873 (50.2)
	Yes	5629 (34.5)	5488 (35.0)
	Missing	2365 (14.5)	2315 (14.8)
Skin-to-skin contact	No	11 533 (70.6)	10 846 (69.2)
	Yes	2662 (16.3)	2729 (17.4)
	Missing	2132 (13.1)	2101 (13.4)
Breast feeding within 1 hour of birth	No	7685 (47.1)	7425 (47.4)
	Yes	4338 (26.6)	4201 (26.8)
	Missing	4304 (26.4)	4050 (25.8)
Oil massaged before placenta came out	No	13 291 (81.4)	12 798 (81.6)
	Yes	724 (4.4)	605 (3.9)
	Missing	2312 (14.2)	2273 (14.5)

Data are presented as mean (SD) for continuous measures and n (%) for categorical measures.

ANC, antenatal care; GA, gestational age; LBW, low birth weight; LMP, last menstrual period; SGA, small for gestational age.

### Intervention coverage

Mothers were visited daily in the first week after delivery to measure adherence to the use of both study oils, with the infants in the mustard seed oil group massaged slightly more times on average than the sunflower seed oil group (table 2). Across the first 7 days, 79% in the mustard seed oil group and 74% in the sunflower seed oil group reported using the assigned study-provided oil at least once per day (table 2). Adherence to the use of study oil was low on the first day of life (4.5% in both groups) but higher on day 2 (75% for sunflower vs 81% for mustard seed oil). Although the study provided oil use was low on the first day, the use of any oil massage was high on this day (98.6%, similar in both randomised groups) (table 3). This question was asked only for the last massage prior to the visit. Thereafter, the adherence was 88% or higher in the sunflower oil group and 93–96% in the mustard seed oil group. The use of any oil massage was above 98% on all days in the first week. Hence, although there was high compliance with study-provided oil, the use of sunflower or mustard seed oil massage was almost universal in the first week of life. Although adherence to the use of study-provided oil was high in both groups, the mustard seed oil group had a very small but consistently higher usage than the sunflower seed oil. This was also the case in the non-study-provided oil usage.

**Table 2:** Adherence to intervention (study-provided oil) during the first 7 days of life

Adherence to study-provided oil (for each child: number of visits when study oil was used/total visits)			
Overall mean per cent (SD)	Mustard oil Mean (SD)	Sunflower (SF) oil Mean (SD)	Mean difference (95% CI): mustard–SF
76.6 (20.4)	79.1 (17.6)	74.0 (22.7)	5.13 (5.61, 4.65)

Adherence to NNIPS oil (each day: total children massaged with NNIPS Study oil/total children visited)				
	Overall (%)	Mustard (%)	SF (%)	Risk ratio (95% CI): SF/mustard
Day 1	4.48	4.40	4.56	1.04 (0.93, 1.15)
Day 2	77.6	80.6	74.6	0.92 (0.91, 0.94)
Day 3	90.7	93.5	87.9	0.94 (0.93, 0.95)
Day 4	93.00	95.3	90.5	0.95 (0.94, 0.96)
Day 5	93.2	96.2	90.0	0.93 (0.93, 0.94)
Day 6	92.1	96.00	88.00	0.92 (0.91, 0.92)
Day 7	92.2	95.5	88.9	0.93 (0.92, 0.94)

NNIPS, Nepal Nutrition Intervention Project-Sarlahi.

**Table 3:** Adherence to any oil massage during the first 7 days of life

Adherence to ANY oil % (each child: no of visits when oil was used/total visits)			
Overall mean (SD)	SF oil Mean (SD)	Mustard oil Mean (SD)	Mean difference (95% CI): SF–mustard
98.88 (7.50)	98.67 (8.36)	99.05 (6.78)	−0.37 (−0.55, –0.19)

Adherence to ANY oil (each day: total children massaged with NNIPS oil/total children visited)				
	Overall (%)	SF (%)	Mustard (%)	Risk ratio (95% CI): SF/mustard
Day 1	98.6	98.3	98.9	0.994 (0.991, 997)
Day 3	99.2	99.1	99.3	0.998 (0.999, 1.000)
Day 7	99.2	99.1	99.3	0.997 (0.995, 0.999)
Day 10	99.0	98.9	99.2	0.998 (0.995, 1.000)
Day 14	98.9	98.6	99.1	0.995 (0.992, 0.997)
Day 21	98.7	98.4	98.9	0.995 (0.992, 0.998)
Day 28	98.5	98.3	98.7	0.995 (0.993, 0.998)

NNIPS, Nepal Nutrition Intervention Project-Sarlahi; SF, sunflower.

### Quality control of oil content

**Figure 2:**
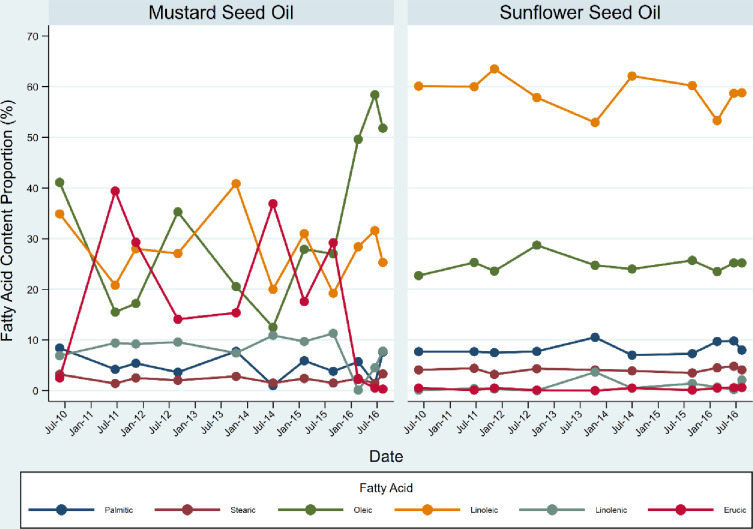
Fatty acid content of mustard and sunflower seed oil over the trial time period.

The fatty acid content of the intervention and control oils was consistent over time (apart from an increase in oleic acid for the last three time points of testing and low erucic acid at the beginning and end of the trial in the mustard seed oil) (figure 2). As expected, primary differences in fatty acid content between intervention and control oils were the higher levels of linoleic acid and lower levels of erucic acid in the sunflower seed oil compared with mustard seed oil.

### Outcome results

NMRs were 31.8/1000 live births in the mustard seed oil group and 30.5/1000 in the sunflower seed oil group (RR=0.95, 95% CI: 0.84, 1.08) (table 4). When stratified by gestational age (a prespecified effect modifier), there was a 12% (not statistically significant) lower mortality in the sunflower seed oil group compared with mustard seed oil among preterm infants (<37 completed weeks) (RR=0.88, 95% CI: 0.74, 1.05). Infants <34 weeks’ gestation had a 17% lower NMR in the sunflower compared with the mustard seed oil group (RR=0.83, 95% CI: 0.67, 1.02). We also examined differences in mortality by birth weight. Infants weighing <2500 g and <1500 g had a 17% higher and 7% lower mortality in the sunflower compared with mustard seed oil group, respectively (RR=1.17, 95% CI: 0.91, 1.50 and RR=0.93, 95% CI: 0.61, 1.41). Causes of death were ascertained through verbal autopsies and assigned causes using the WHO algorithm. There were no differences in cause of death by treatment groups, although the study was not powered to look at such differences.^17^ Rates of possible severe infection in neonates were 13.7/100 live births in the mustard seed oil group and 13.5/100 in the sunflower seed oil group (RR=0,98, 95% CI: 0.92, 1.04). There were no differences in possible severe infections between treatment groups when stratified by gestational age or birth weight.

**Table 4:** Neonatal mortality and possible severe infection by intervention group and stratified by gestational age and birth weight

Mortality within 28 days	Mustard seed oil			Sunflower (SF) seed oil			RR (SF/mustard)	95% CI
	Live births	Deaths	Rates (per 1000)	Live births	Deaths	Rates (per 1000)		
All infants	16 327	520	31.8	15 676	478	30.5	0.95	0.84, 1.08
By gestational age								
≥37 weeks	13 793	291	21.1	13 301	290	21.8	1.03	0.87, 1.21
<37 weeks	2533	229	90.4	2373	188	79.2	0.88	0.74, 1.05
<34 weeks	728	158	217.0	649	117	180.3	0.83	0.67, 1.02
By birth weight*								
≥2500 g	8612	82	9.5	8307	58	7.0	0.74	0.53, 1.03
<2500 g	3713	113	30.4	3538	126	35.6	1.17	0.91, 1.50
<1500 g	51	23	450	62	26	419	0.93	0.61, 1.41

Possible severe infection	Mustard seed oil			SF seed oil			RR (SF/mustard)	95% CI
	Live births	Cases	Rates (per 100)	Live births	Cases	Rates (per 100)		
All infants	14 145	1940	13.7	13 483	1823	13.5	0.98	0.92, 1.04
By gestational age								
≥37 weeks	11 954	1618	13.5	11 466	1506	13.1	0.97	0.90, 1.03
<37 weeks	2191	322	14.7	2015	317	15.7	1.07	0.93, 1.23
<34 weeks	627	116	18.5	537	101	18.8	1.01	0.79, 1.29
By birth weight*								
≥2500 g	8307	1055	12.7	8694	1159	13.3	0.95	0.88, 1.03
<2500 g	3538	550	15.5	3713	541	14.5	1.07	0.96, 1.19
<1500 g	62	27	43.5	51	19	37.2	1.17	0.74, 1.84

*Weight taken within 72 hours of birth.

RR, relative risk.

## Discussion

We did not find any impact of switching from traditional mustard to sunflower seed oil massage of neonates on NMR or possible bacterial infections. There was some suggestion from the point estimates that there might be benefit for preterm and moderately preterm infants, although these comparisons were not statistically significant. This is in line with findings of NMR benefit in hospital-based studies^8^ that were conducted specifically in preterm infants, but not with reduction in infections in preterm infants in those hospital settings.^4–7^ It is possible that our definition of infection (based on parental recall) was not as accurate as those used in hospital-based studies that used physical examination and blood work. This would bias the RR towards a null result if random misclassification were present. Compared with the impact on NMR in one community-based randomised community trial, our results are quite similar (no impact on NMR overall in the intent-to-treat analysis but a reduction in NMR among very preterm babies as measured by <1500 g in the trial in Uttar Pradesh,^15^ and <34 weeks’ gestation in our trial). However, the impact was much larger in the very low birthweight babies in the Indian trial (52% reduction) than we saw in the moderately preterm infants (17% reduction). Our analysis of impact stratified by <1500 g did not show an impact on mortality (7% reduction), which is different from the Uttar Pradesh trial.

In general, the adherence to daily massage with study-provided oils was high and comparable across the two groups. However, on the first day of life, the adherence to study-provided oil was very low in both groups. This may be due to the aim of visiting soon after birth on the first day of life. Hence, this measure is likely not a good reflection of the adherence for the first full 24 hours of life. However, maternal report of the use of non-study oil for massage was 98.6% on the first day (asking about the first 24 hours). The use of non-study oils could have modified the outcomes of interest, given the importance of oil massage on the first day of life.^18^ Given our aim of arriving soon after birth, we were unable to fully measure oil use in the first 24 hours of life. There were some important differences between our trial and the one in India. In India, the intervention of gentle massage three times a day with sunflower seed oil without the addition of abrasive treatment by adding grain to the oil was compared with a group that practised usual care in the community without any messaging around gentle massage. There was a lower baseline NMR in Nepal than India (50/1000 live births compared with 30/1000 in Nepal), higher frequency of massage reported in Nepal than in India, a lower proportion of births in Nepal occurred in facilities than in India and there was a higher level of adherence in Nepal than in India. In Nepal, we also provided mustard seed oil to study participants (the Indian trial provided only the sunflower seed oil, with the mustard seed oil group obtaining their oil as usual in the absence of the trial). In the Indian trial, there were two postpartum visits, whereas there were seven in the Nepal trial. This may have improved adherence in the Nepal trial relative to the one in India.

Although a community-based trial, our intent was to measure efficacy and attention was paid to providing oil of similar purity of both kinds to mothers and to encourage use of study-provided oils to both groups with regular visits by female study staff. Oil massage was universal and practised three to five times per day in both groups. No attempt was made to alter the way in which the massage was done, which could have been vigorous and potentially damaging to the vernix and to the stratum corneum, the outermost layer of the skin and an important part of the skin barrier, especially among preterm infants. In a subgroup of infants followed for skin function in our study, we found no difference in erythema, rash, skin dryness and transepidermal water loss by oil group but a more rapid decrease in skin surface acidity pH in the sunflower seed oil group.^19^ A more rapid acid mantle increase observed in the sunflower seed oil group may be protective in neonates, especially preterm infants. However, our substudy found no difference in pH by gestational age, which was the group in which we saw the most reduction in mortality but not in infection with 80% power.

We did observe an instability of oleic and erucic acids in the mustard seed oil over time in comparison with sunflower seed oil. Oleic acid ranged from 16% to 68% for mustard vs ~22–29% in sunflower seed oil. Erucic ranged from 0% for one period to 39% for another, although levels were 3% or below for four periods. Highly variable levels of erucic acid (2.7–41.4%) were found in mustard oil samples from Bangladesh in the study of oil effects on the skin barrier of a mouse model of infant skin, but no erucic acid was detected in the sunflower seed oil.^12^ The mustard and sunflower seed oils used in our trial came from a factory where quality was checked by the government of Nepal prior to sale. Both oils were kept in hygienic conditions at room temperature until dispersed to participants. The source of the observed variability is unknown but may be an inherent property of mustard relative to sunflower seed oil. Sawicka *et al* reported erucic levels at 3.8% and 22.2% from two cultivars of black mustard.^20^ Oral consumption of erucic acid was associated with the development of alopecia and scaly skin lesions but topical application was not discussed.^21^ A cosmetic ingredient review of the safety of 102 fatty acids (including erucic acid) in 2019 deemed them to be safe for cosmetics whereby their levels are within non-irritating and non-sensitising ranges. Perhaps the stability of the acid profile of sunflower seed oil, in addition to its high linoleic acid content, may be arguments for its use on skin of newborns.

Oils with erucic acid levels over 2% have been banned in the USA and Europe since 2019 because of safety concerns with ingestion. While fatty acids are absorbed through the skin, there is no evidence linking safety concerns from oral ingestion with absorption through the skin of preterm neonates at this time.

Given the simplicity of this intervention (no change to the behaviour of neonatal oil massage, only a change of oil type, both of which have the same retail price), this would have had programmatic value if it had shown a health impact. Our aim was to make this a universal recommendation rather than target specific high-risk infants such as those born preterm. While there is some dose–response indication that more severely preterm infants might benefit from this intervention, our trial was not powered to detect the difference we observed with 80% power.

Given the state of evidence prior to results from this study being available, the WHO recommended in their care of the preterm or LBW baby that ‘Application of topical oils to the body of preterm or LBW infants may be considered (conditional recommendation, moderate-certainty evidence). The Guideline Development Group (GDG) noted that there were limited data on the type, dose, timing of initiation and duration of oil use. Based on most of the trials included in the evidence review, the GDG suggested that sunflower or coconut oils may be used and that initiation and duration of use may be based on clinical judgement. The GDG also felt that application of oils should be done gently to avoid disrupting skin integrity.’ No discussion of harm from mustard oil was discussed. Given this recommendation and results of the Indian and Nepal trials, there is increasing evidence against the use of mustard oil, but we do not believe enough to recommend against its use at this time.^22^

## Conclusions

There was no overall impact on neonatal mortality or possible bacterial infections by switching from mustard to sunflower seed oil for neonatal massage. The data indicate a possible survival benefit among preterm and especially moderate to very preterm or very small babies, but this trial was not powered to detect such benefits. Given the simplicity and low cost of such an intervention, further evaluation of switching from mustard to sunflower seed oil massage of preterm and very small neonates in a community-based setting with appropriate sample size may be warranted.

## Data Availability

Data are available upon reasonable request. Data are available upon reasonable request or through Synapse.
