# Supplementary material for: Impact of topical applications of sunflower seed oil on neonatal mortality and morbidity in southern Nepal: a community-based, cluster-randomised trial
Source: BMJ Glob Health. 2024 Feb 29;9(2):e013691. doi: 10.1136/bmjgh-2023-013691 (PMC10910473; doi:10.1136/bmjgh-2023-013691)
Supplement: Supplementary data [file bmjgh-2023-013691supp001.pdf]

Study conceptualisation	1. How does this study address local research and policy priorities?<br><br>Use of mustard seed oil massage for newborns is a ubiquitous practice in Nepal. Sunflower seed oil is available and costs the same. If sunflower seed oil has improved health outcomes for newborn babies, this would be an important finding on which the ministry of health could act to change behavior. This addresses a local priority to reduce neonatal deaths and improve neonata health.<br><br>2. How were local researchers involved in study design?<br><br>Collaborators from the Institute of Medicine (Tribhuvan University) were our partners in this research and engaged in study design as it was determined. In addition, Dr. Subarna Khatry, the NNIPS director, was also engaged in study design.
Research management	1. How has funding been used to support the local research team(s)?<br><br>Support for level of effort of salaries or consulting fees were used to support the local research team.
Data acquisition and analysis	1. How are research staff who conducted data collection acknowledged?<br><br>Authorship is provided to those contributing to the science and the conduct of research.<br><br>2. How have members of the research partnership been provided with access to study data?<br><br>Study data is available to all research partners.<br><br>3. How were data used to develop analytical skills within the partnership?<br><br>Primary data analysis was conducted by Seema Subedi, a Nepali citizen residing Nepal but employed by Johns Hopkins University.
Data interpretation	1. How have research partners collaborated in interpreting study data?<br><br>All partners reviewed the analyses and were asked to comment.
Drafting and revising for intellectual content	1. How were research partners supported to develop writing skills?<br><br>The manuscript was drafted by two authors but all authors were asked to provide comments and feedback on the writing.<br><br>2. How will research products be shared to address local needs?<br><br>Meetings were held annually with national and local stakeholders (two separate committees mandated by the government of Nepal) to explain the study and obtain feedback. Once these results are published, we plan

	to present results to these groups.
Authorship	1. How is the leadership, contribution and ownership of this work by LMIC researchers recognised within the authorship?<br><br>Those who contributed scientifically, with study design, organization, and conduct, have been acknowledged through authorship.<br><br>2. How have early career researchers across the partnership been included within the authorship team<br><br>Junior researchers have been included in the authorship team.<br><br>3. How has gender balance been addressed within the authorship?<br><br>We believe we have gender balance within our authorship. This was naturally attained based on roles of each author that merited authorship.
Training	1. How has the project contributed to training of LMIC researchers?<br><br>Some LMIC researchers are quite senior. One is more junior and this project contributed to her training. We also had a recently graduated LMIC bachelor's student who worked on this project, and an LMIC masters student worked as an intern.
Infrastructure	1. How has the project contributed to improvements in local infrastructure?<br><br>The project has trained many local men and women (mostly women) to conduct consents and interview female participants, as well as do anthropometry and other physical measurements.
Governance	1. What safeguarding procedures were used to protect local study participants and researchers?<br><br>This study was overseen by the Institute of Medicine Tribhuvan University IRB. A group from the Social Welfare Council of the Government of Nepal also visited and oversaw activities of the study.
